# Phagocytosis: Our Current Understanding of a Universal Biological Process

**DOI:** 10.3389/fimmu.2020.01066

**Published:** 2020-06-02

**Authors:** Eileen Uribe-Querol, Carlos Rosales

**Affiliations:** ^1^División de Estudios de Posgrado e Investigación, Facultad de Odontología, Universidad Nacional Autónoma de México, Mexico City, Mexico; ^2^Departamento de Inmunología, Instituto de Investigaciones Biomédicas, Universidad Nacional Autónoma de México, Mexico City, Mexico

**Keywords:** immunoglobulin, antibody, phagocytosis, neutrophil, ERK, complement, integrin

## Abstract

Phagocytosis is a cellular process for ingesting and eliminating particles larger than 0.5 μm in diameter, including microorganisms, foreign substances, and apoptotic cells. Phagocytosis is found in many types of cells and it is, in consequence an essential process for tissue homeostasis. However, only specialized cells termed professional phagocytes accomplish phagocytosis with high efficiency. Macrophages, neutrophils, monocytes, dendritic cells, and osteoclasts are among these dedicated cells. These professional phagocytes express several phagocytic receptors that activate signaling pathways resulting in phagocytosis. The process of phagocytosis involves several phases: i) detection of the particle to be ingested, ii) activation of the internalization process, iii) formation of a specialized vacuole called phagosome, and iv) maturation of the phagosome to transform it into a phagolysosome. In this review, we present a general view of our current understanding on cells, phagocytic receptors and phases involved in phagocytosis.

## Introduction

Phagocytosis is a basic process for nutrition in unicellular organisms, and it is also found in almost all cell types of multicellular organisms. However, only a specialized group of cells called professional phagocytes (1) accomplish phagocytosis with high efficiency. Macrophages, neutrophils, monocytes, dendritic cells, and osteoclasts are among these dedicated cells. Professional phagocytes are responsible of removing microorganisms and of presenting antigens to lymphocytes in order to activate an adaptive immune response. Fibroblasts, epithelial cells, and endothelial cells can also accomplish phagocytosis with low-efficiency and are thus described as non-professional phagocytes. These cells cannot ingest microorganisms, but are important in eliminating dead cells and maintaining homeostasis (2). Phagocytosis is the process of sensing and taking in particles larger than 0.5 μm. The particle is internalized into a distinctive organelle, the phagosome. This phagosome subsequently changes the structure of its membrane and the composition of its contents in a process known as phagosome maturation (3). The phagosome next fuses with lysosomes to become a phagolysosome. This new organelle contains enzymes that can degrade the ingested particle (4).

Phagocytes can identify several diverse particles that could potentially be ingested, including apoptotic cells and microbial pathogens. Discrete receptors mediate this recognition by sensing the particle as a target and then initiating signaling pathways that favor phagocytosis. Plasma membrane receptors of phagocytes are divided into non-opsonic or opsonic receptors. Non-opsonic receptors directly identify distinct molecular patterns on the particle to be ingested. These receptors include C-type lectins, such as Dectin-1 (5), Dectin-2, Mincle, or DC-SIGN (6); lectin-like recognition molecules, such as CD33; and scavenger receptors (7). Although, the toll-like receptors (TLRs) (8) can also detect molecular patterns on pathogens, they are not phagocytic receptors. Nevertheless, TLRs can cooperate with phagocytic receptors to make phagocytosis more efficient (9). Opsonic receptors detect host-derived proteins bound to target particles. These proteins known as opsonins include antibodies, fibronectin, complement, milk fat globulin (lactadherin), and mannose-binding lectin (10). Opsonins label particles as targets of phagocytosis. Fc receptors (FcR) and the complement receptors (CR) are the best characterized opsonic receptors. FcRs bind to the Fc portion of IgG (11, 12) or IgA antibodies (13). Complement receptors bind to activated complement components, such as iC3b, deposited on the particle (14).

Upon binding to the particle, phagocytic receptors initiate signaling pathways leading to remodeling of the actin cytoskeleton and lipids in the membrane, that result in the membrane extending to cover the particle (15). Then, the membrane closes at the distal end creating the phagosome. Thus, the particle gets internalized inside the phagosome. During membrane extension, the phagocytic receptors bind to the target in a sequential order and help completing the formation of the phagosome (16, 17). Next, this early phagosome undergoes sequential fusion and fission events with endocytic vesicles to create a late phagosome (18). This late phagosome then fuses with lysosomes and becomes a phagolysosome. The process to change a phagosome into a potent anti-microbial phagolysosome is known as phagosome maturation (3).

The process of phagocytosis involves several phases: (i) detection of the particle to be ingested, (ii) activation of the internalization process, (iii) formation of a specialized vacuole called phagosome, and (iv) phagosome maturation. In this review, we present the main phagocytic receptors and a general view of our current understanding on phagocytosis.

## Detection of the Target Particle

The first phase in phagocytosis is the detection of the target particle. Detection is mediated by dedicated receptors on phagocytic cells. Receptors directly recognizing pathogen-associated molecular patterns (PAMPs) are the pattern-recognition receptors (PRRs). Some of these PRRs can initiate phagocytosis and thus constitute the non-opsonic receptors for phagocytosis. Other PRRs, for example TLRs, can bind to PAMPs but not induce phagocytosis. These receptors however, can prepare (prime) the cell for phagocytosis. Foreign particles can also be detected indirectly by opsonic receptors. The receptors for antibody and complement are the best described opsonic receptors.

### Non-opsonic Receptors

#### Receptors for Microorganisms

Some receptors that directly bind PAMPs and can induce phagocytosis include Dectin-1, Mincle, MCL, and DC-SIGN (Table 1). All these molecules are members of the family of C-type lectin receptors (6). Dectin-1 (dendritic cell-associated C-type lectin-1) recognizes yeast polysaccharides (19), and it has been shown to be a bona fide phagocytic receptor. When expressed on non-phagocytic heterologous cells, Dectin-1 allowed the cells to perform phagocytosis (19–21). *In vivo*, it is also possible that Dectin-1 cooperates with other phagocytic receptors in particular cells. For example, in neutrophils, Dectin-1 has been reported to connect to the phagocytic receptor Mac-1 (CD11b/CD18, CR3) (33). Mincle (macrophage-inducible C-type lectin) is a receptor for trehalose dimycolate (TDM), which is present on the cell wall of some mycobacterium (22). MCL (macrophage C-type lectin, Dectin-3) is another receptor for TDM that also binds α-mannans. Both, Mincle and MCL are considered bona fide phagocytic receptors, because when individually expressed in 293T cells, they induce internalization of beads covered with antibodies against each receptor (23). In myeloid cells, Mincle and MCL seem to cooperate for enhanced phagocytosis by forming heterodimers on the cell membrane (23). DC-SIGN (dendritic cell-specific ICAM-3-grabbing non-integrin) is another receptor that can bind multiple microbial pathogens, including viruses, fungi, and bacteria (6), through recognition of fucosylated glycans and mannose-rich glycans (24). DC-SIGN was shown to be a phagocytic receptor by expressing it in non-phagocytic human myeloid K562 cells or in epithelial HeLa cells. K562 cells were then capable of internalizing *Mycobacterium tuberculosis* mannose-capped lipoarabinomannan (ManLAM)-coated beads (25), while HeLa cells could bind and internalize *Escherichia coli* bacteria (26). DC-SIGNR is another C-type lectin receptor with high homology to DC-SIGN, and capable of binding mannose-rich ligands (34). Therefore, DC-SIGNR is also very likely a phagocytic receptor. Other C-type lectin domain-containing proteins have been implicated in phagocytosis long before Dectin-1 and other C-type lectin receptors (6). The macrophage mannose receptor (CD206) presents several C-type lectin carbohydrate recognition domains, which detect α-mannan on many microorganisms (Table 1). The mannose receptor was also shown to be a bona fide phagocytic receptor when expressed in non-phagocytic COS-1 cells. Transfected COS-1 cells were then able to mediate internalization of zymosan (27).

**Table 1:** Human non-opsonic phagocytic receptors and their ligands.

**Receptor**	**Ligands**	**Reference(s)**
**Non-opsonic receptors**		
Dectin-1	Fungal beta-glucan Polysaccharides of some yeast cells	(19–21)
Mincle	Trehalose dimycolate of Mycobacteria	(22, 23)
MCL	Trehalose dimycolate α-Mannan	(23)
DC-SIGN	Fucosylated glycans Mannose-rich glycans	(24–26)
Mannose receptor	Mannan	(27)
CD14	Lipopolysaccharide-binding protein	(28)
Scavenger receptor A	Lipopolysaccharide, lipoteichoic acid	(29, 30)
CD36	*Plasmodium falciparum*-infected erythrocytes	(31)
MARCO	Bacteria	(32)

Other PAMP receptors are also involved in phagocytosis, but it is still not clear whether they can induce phagocytosis on their own, or they do it indirectly by just bringing the particle close to the phagocyte (35). It is also possible that these receptors just prime the phagocyte, while other receptors mediate phagocytosis (35). CD14, scavenger receptor A (SR-A), CD36, and MARCO are among these receptors (Table 1). CD14 is a receptor for lipopolysaccharide (LPS)-binding protein (28). SR-A recognizes LPS on Gram-negative bacteria (29), and on *Neisseria meningitidis* (30). CD36 detects *Plasmodium falciparum*-infected erythrocytes (31), and MARCO (macrophage receptor with collagenous structure) is involved in recognition of several bacteria (32).

#### Receptors for Apoptotic Cells

In multicellular organisms many cells die constantly by apoptosis for maintaining homeostasis. These apoptotic cells are eliminated by phagocytosis. Detection of apoptotic cells requires particular receptors for molecules that only appear on the membrane of dying cells. These molecules include lysophosphatidylcholine, and phosphatidyl serine (PS) (36). These molecules deliver to phagocytes an “eat me” signal (37). Receptors directly recognizing PS include TIM-1, TIM-4 (38), stabilin-2 (39), and BAI-1 (brain-specific angiogenesis inhibitor 1) (40) (Table 2). The integrin αvβ3 can also bind PS after other receptors, for example lactadherin, connect PS to the integrin (41). The integrin α_V_β5 (42), CD36 (45), and CD14 (44, 46) are also receptors for apoptotic cells (Table 2). Some normal cells, for example activated B and T lymphocytes, may express significant levels of PS on their surface. These cells avoid phagocytosis by expressing at the same time molecules that serve as “don't eat me” signals (2). One such molecule is CD47, a ligand to the receptor SIRPα (signal regulatory protein α), which is expressed on phagocytes (47). Upon engagement, SIRPα delivers an inhibitory signal for actin assembly (47). The signaling events from these receptors to activate phagocytosis are just beginning to be elucidated. Since phagocytosis of apoptotic cells is central to homeostasis (48), determining the phagocytosis mechanisms of all these receptors for apoptotic cells will be an active area of future research.

**Table 2:** Receptors for apoptotic cells.

**Receptor**	**Ligands**	**Reference(s)**
TIM-1^*^	Phosphatidylserine	(38)
TIM-4^*^	Phosphatidylserine	(38)
Stabilin-2	Phosphatidylserine	(39)
BAI-1^*^	Phosphatidylserine	(40)
Lactadherin and α_V_β3	MFG-E8^*^	(41)
α_V_β5	Apoptotic cells	(42)
CD36	Oxidized lipids	(43)
CD14	Phosphatidylserine (?)	(44)

* *TIM, T cell immunoglobulin mucin; BAI-1, brain-specific angiogenesis inhibitor 1; MFG, milk fat globule*.

### Opsonic Receptors

Foreign particles can also be labeled for phagocytosis by opsonins, which are host-derived proteins that bind specific receptors on phagocytic cells. Important opsonins promoting efficient phagocytosis include antibody (IgG) molecules and complement components. These opsonins and their receptors are the best studied so far (Table 3).

**Table 3:** Human opsonic phagocytic receptors and their ligands.

**Receptor**	**Ligands**	**Reference(s)**
FcγRI (CD64)	IgG1 = IgG3 > IgG4	(49)
FcγRIIa (CD32a)	IgG3 ≥ IgG1 = IgG2	(49)
FcγRIIIa (CD16a)	IgG	(49)
FcαRI (CD89)	IgA1, IgA2	(13, 50)
CR1 (CD35)	Mannan-binding lectin, C1q, C4b, C3b	(51)
CR3 (α_M_β2, CD11b/CD18, Mac-1)	iC3b	(52)
CR4 (α_V_β2, CD11c/CD18, gp190/95)	iC3b	(52)
α5β1 (CD49e/CD29)	Fibronectin, vitronectin	(53)

#### Fcγ Receptors

**Figure 1:**
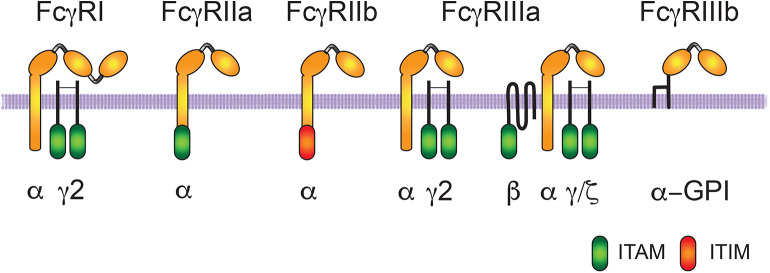
Human Fcγ receptors. The human receptors for the Fc portion of immunoglobulin G (IgG) molecules are classified in three groups FcγRI, FcγRII, and FcγRIII. The IgG binding α-subunit in the high affinity FcγRI, possesses three immunoglobulin (Ig)-like extracellular domains. The α-subunit in the other low-affinity receptors presents only two Ig-like domains. Activating receptors contain an ITAM (immunoreceptor tyrosine-based activation motif) sequence within the α subunit (for FcγRIIa) or within the accessory γ and ζ chains (for FcγRI and FcγRIIIa). FcγRIIIa has a homodimer of γ chains in macrophages, natural killer (NK) cells, and dendritic cells, whereas it has a heterodimer of γ/ζ chains and an extra β chain in basophils and mast cells. FcγRIIIb is also an activating receptor, which is bound to the cell membrane via a glycosylphosphatidylinositol (GPI) anchor. In contrast, FcγRIIb is an inhibitory receptor containing an ITIM (immunoreceptor tyrosine-based inhibition motif) sequence.

Fcγ receptors (FcγR) are glycoproteins that specifically bind the Fc part of IgG molecules (12, 54). When FcγR engage IgG molecules in multivalent antigen-antibody complexes, they get clustered on the membrane of the cell, and then trigger phagocytosis as well as other cellular responses (11, 55) (Figure 1).

Three types of FcγR are expressed on human cells, FcγRI (CD64), FcγRII (CD32), and FcγRIII (CD16) (56) (Figure 1). FcγRI has three Ig-like domains, and displays high affinity for IgG molecules. In contrast, FcγRII and FcγRIII have two Ig-like domains, and display low-affinity for IgG molecules. Thus, they can only bind multimeric immune complexes (57). FcγRI is expressed together with a dimer of the common Fc receptor gamma (FcRγ) chain. Each FcRγ chain contains tyrosine residues within an immunoreceptor tyrosine-based activation motif (ITAM; consensus sequence: YxxI/Lx_(6−12)_YxxI/L) (58, 59). The clustering of activating FcγRs results in the phosphorylation of tyrosine residues in the ITAM sequence present within the cytoplasmic domain of the receptor (as is the case with FcγRIIa and FcγRIIc), or in an associated FcR common γ-chain (as with FcγRI and FcγRIIIa) (11, 12, 57). These tyrosine residues are phosphorylated upon activation and are essential for receptor signaling. FcγRII presents two isoforms: FcγRIIa expressed mainly in phagocytic cells and FcγRIIb expressed mainly in B lymphocytes (56). FcγRIIa does not associate with FcRγ chains, but has an ITAM motif in its cytoplasmic tail. FcγRIIb also does not associate with FcRγ chains, but in contrast, has an immunoreceptor tyrosine-based inhibition motif (ITIM; consensus sequence: S/I/V/LxYxxI/V/L) in its cytoplasmic tail involved in negative signaling (60). Phosphorylated tyrosine residues within the ITIM recruit phosphatases that down-modulate signals coming from ITAM-containing activated receptors (60, 61). FcγRIIb functions as a negative regulator of cell functions, such as phagocytosis (62, 63). FcγRIII presents two isoforms: FcγRIIIa expressed in macrophages, natural killer (NK) cells, basophils, mast cells and dendritic cells, and FcγRIIIb expressed exclusively on neutrophils (57) (Figure 1). FcγRIIIa is a receptor with a transmembrane portion and a cytoplasmic tail, associated with a dimer of FcRγ chains, while FcγRIIIb is a glycosylphosphatidylinositol (GPI)-linked receptor, lacking a cytoplasmic tail and no known associated subunits (64) (Figure 1).

#### Complement Receptors

**Figure 2:**
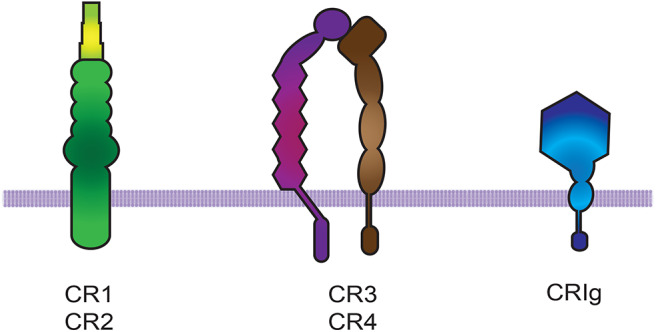
Complement receptors. There are three groups of complement receptors: (i) the short consensus repeat (SCR) modules that code for CR1 and CR2, (ii) the β2 integrin family members CR3 and CR4 (66), and (iii) the immunoglobulin Ig-superfamily member CRIg.

Complement receptors (CRs) bind activated complement molecules deposited on microorganisms or cells (65, 66). Complement receptors belong to three groups of molecules: (i) CR1 and CR2, which are formed by short consensus repeat (SCR) elements, (ii) CR3 and CR4, which belong to the β2 integrin family (66), and (iii) CRIg, which belongs to the immunoglobulin Ig-superfamily (14) (Figure 2). The integrin α_M_β2 (also known as CD11b/CD18, CR3, or Mac-1) binds the complement component iC3b, and is the most efficient phagocytic receptor among complement receptors (66–68).

### Phagocytic Receptors Cooperation

For efficient recognition of the target particle and initiation of phagocytosis, numerous receptors on the phagocyte membrane must interact with several IgG molecules on the opsonized particle. For this, receptors must have good mobility of the membrane (69) so that they can aggregate and get activated. However, free diffusion is not easy for most phagocytic receptors, because they are among other (usually bigger) transmembrane glycoproteins that cover the cell surface. Phagocytic receptors are very short molecules compared to these longer glycoproteins; hence short receptors are obscured among a layer of large glycoproteins (the glycocalyx), such as mucins, hyaluronan, and the membrane phosphatases CD45 and CD148 (70). In addition, many large glycoproteins are tied to the cytoskeleton, and can interfere with the lateral diffusion of receptors on the cell membrane (15, 17).

**Figure 3:**
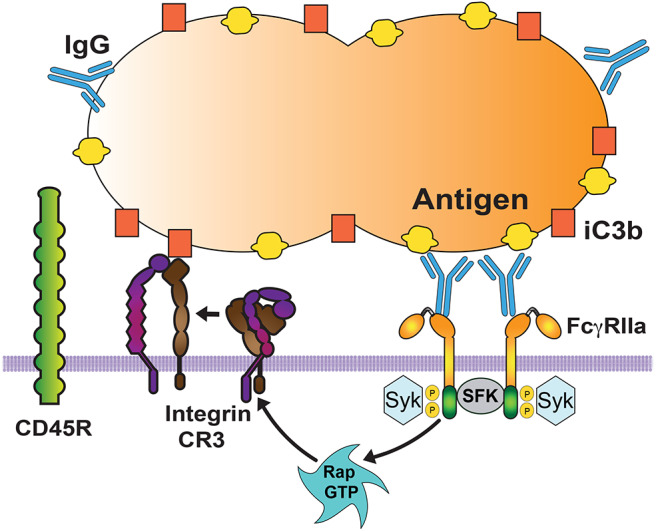
Cooperation among phagocytic receptors. Most phagocytic receptors, such as receptors for antibody (FcγRIIa) and receptors for complement (Integrin CR3) cooperate to bind the particle to be ingested. FcγR aggregation triggers an inside-out signal that activates integrins via the GTPase Rap. Activated Rap (Rap GTP) is responsible for integrin activation. Then, activated integrins also bind to the particle (via complement fragment C3b), and form a diffusion barrier that excludes larger molecules, such as the transmembrane phosphatase CD45. This allows other Fc receptors to be engaged and increase the signaling for phagocytosis. SFK, Src family kinases. Syk, spleen tyrosine kinase.

Interactions of Fcγ receptors with possible targets can be enhanced by cooperation with other receptors that can remove larger glycoproteins from the area of the membrane in contact with the target particle. The result is that Fcγ receptors can then diffuse more freely on the membrane and engage more IgG molecules (16) (Figure 3). Removal of large glycoproteins from the membrane area of contact with the target particle is achieved by activated integrins. Integrins, for example CR3, increase their affinity for their ligand after they receive an inside-out signal (71, 72) from other receptors such as Fc receptors (73), TLRs (74), or CD44 (75). Inside-out signaling leads to activation of integrins (66, 76) via the small GTPase Rap1 (77). Activated integrins extend their conformation and create a diffusion barrier that keeps larger glycoproteins, for example the phosphatase CD45, away from phagocytic receptors (16) (Figure 3). Also, extended integrins can engage more distant ligands on the particle (78) and create a progressive wave of large molecules migrating in front of the bound Fcγ receptors, which aggregate in microclusters to mediate a strong adhesion between the phagocyte membrane and the particle to be ingested (17). Thus, during phagocytosis integrins cooperate with Fcγ receptors by promoting adhesion to the opsonized particle (79). Interestingly, this type of cooperation was implied by earlier studies showing that in neutrophils FcγRIIIb associates with Mac-1 integrins (80, 81).

## Activation of the Internalization Process

When a particle is recognized by phagocytic receptors, various signaling pathways are activated to initiate phagocytosis. Reorganization of the actin cytoskeleton and changes in the membrane take place resulting in a depression of the membrane area touching the particle, the phagocytic cup. Then, pseudopods are formed around the particle until the membrane completely covers the particle to form a new phagosome inside the cell. The signaling mechanisms to activate phagocytosis are best-known for Fc receptors and for complement receptors (10, 67, 82–84). For other phagocytic receptors, signaling pathways are just beginning to be investigated.

### Fcγ Receptor Signaling

**Figure 4:**
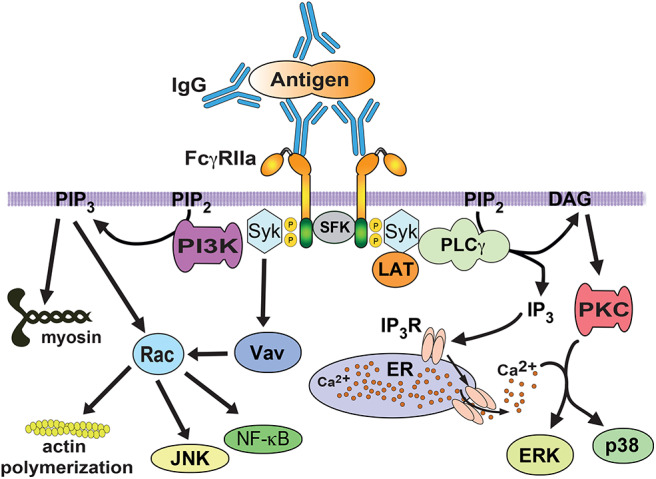
FcγR signaling for phagocytosis. FcγRIIa crosslinking by immunoglobulin (IgG) bound to a particle, induces activation of Src family kinases (SFK), which phosphorylate tyrosine residues in the ITAM sequence within the cytoplasmic tail of the receptor. Then, spleen tyrosine kinase (Syk) associates with phosphorylated ITAMs and leads to phosphorylation and activation of a signaling complex formed by the scaffold protein LAT (linker for activation of T cells) interacting with various proteins. One of these proteins is phospholipase C gamma (PLCγ), which produces inositoltrisphosphate (IP_3_), and diacylglycerol (DAG). These second messengers cause calcium release and activation of protein kinase C (PKC), respectively. PKC leads to activation of extracellular signal-regulated kinases (ERK and p38). The guanine nucleotide exchange factor Vav activates the GTPase Rac, which is involved in regulation of the actin cytoskeleton. Rac is also involved in activation of transcription factors such as NF-κB and JNK. The enzyme phosphatidylinositol 3-kinase (PI3K), which is recruited and activated by Syk, generates the lipid phosphatidylinositol-3,4,5-trisphosphate (PIP_3_) at the phagocytic cup. This lipid also regulates Rac activation, and contractile proteins such as myosin. P represents a phosphate group. ER, endoplasmic reticulum. IP_3_R, receptor (calcium channel) for inositoltrisphosphate.

Fcγ receptors get activated when they bind to antibody molecules covering the target particle and get clustered on the phagocyte membrane. Upon clustering of Fcγ receptors, they co-localize with Src-family kinases (such as Lyn, Lck, and Hck). These kinases phosphorylate tyrosines within the ITAM. Then, Syk (spleen tyrosine kinase) binds to the phosphorylated ITAMs and gets activated (67, 85). Activated Syk, in turn, can phosphorylate multiple substrates and initiate different pathways that connect to distinct cellular responses such as phagocytosis (67, 85, 86) and transcriptional activation (86) (Figure 4). Important Syk substrates involved in phagocytosis are the adaptor molecule LAT (linker for activation of T cells), phosphatidylinositol 3-kinase (PI 3-K), and phospholipase Cγ (PLCγ) (87, 88) (Figure 4). Phosphorylation of LAT induces docking of additional adaptor molecules such as Grb2 and Gab2 (Grb2-associated binder 2) (89). Phosphorylated (active) PI 3-K generates the lipid phosphatidylinositol-3,4,5-trisphosphate (PIP_3_) at the phagocytic cup (90, 91). This lipid also regulates activation of the GTPase Rac, and contractile proteins such as myosin. Active Rac is important in actin remodeling and activation of other signaling molecules such as JNK and the nuclear factor NF-κB (Figure 4). Activated PLCγ produces inositoltrisphosphate (IP_3_), and diacylglycerol (DAG). These second messengers cause calcium release and activation of protein kinase C (PKC), respectively (92). PKC leads to activation of extracellular signal-regulated kinases (ERK and p38) (93). The Guanine nucleotide exchange factor (GEF) Vav activates GTPases of the Rho and Rac family (94), which are involved in regulation of the actin polymerization that drives pseudopod extension (Figure 4).

### Complement Receptor Signaling

**Figure 5:**
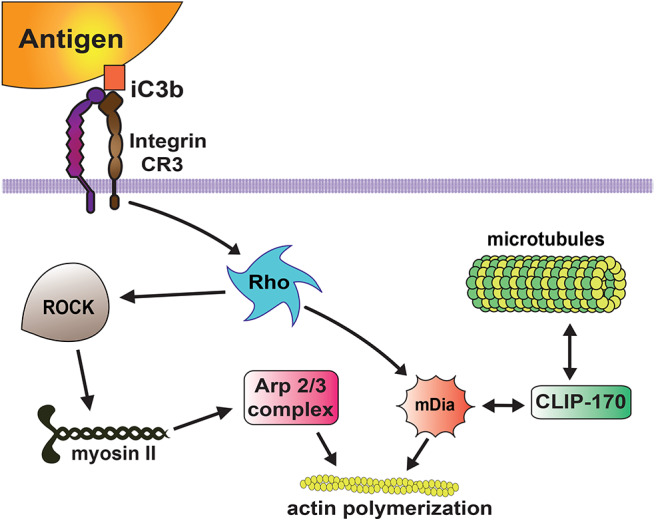
Complement receptor signaling for phagocytosis. The complement receptor 3 (CR3 integrin) binds the complement molecules (iC3b) deposited on the target particle, and activates a signaling pathway that leads to activation of the GTPase Rho. Then, active Rho induces actin polymerization via two mechanisms. Rho activates Rho kinase (ROCK), which phosphorylates and activates myosin II, inducing accumulation of Arp2/3 and actin assembly at the phagocytic cup. Rho also promotes accumulation of mDia1 (mammalian diaphanous-related formin 1), which stimulates linear actin polymerization. In addition, mDia1 binds directly to the microtubule-associated protein CLIP-170 providing a link to the microtubule cytoskeleton.

Among complement receptors, CR3 (integrin Mac-1) is the most efficient phagocytic receptor (66, 67). From very early studies, it has been realized that CR3 on macrophages initiates a different type of phagocytosis from the one mediated by antibody Fcγ receptors. CR3-mediated phagocytosis is characterized by “sinking” of the target particle into the cell membrane without generation of pseudopods around the particle (95). Also, the usage of cytoskeleton components for particle internalization is different between FcγR- and CR-mediated phagocytosis. During FcγR-mediated phagocytosis the actin cytoskeleton is used, whereas during CR-mediated phagocytosis the actin and microtubule cytoskeletons are involved (96, 97). In complement phagocytosis F actin remodeling depends on activation of the GTPase Rho, but not on the GTPases Rac or Cdc42 (98, 99). Active Rho in turn, promotes actin polymerization via two mechanisms (Figure 5). First, Rho stimulates Rho kinase, which phosphorylates and activates myosin II (100). Myosin then leads to activation of the Arp2/3 complex, which promotes actin assembly at the phagocytic cup (100). Second, Rho can induce accumulation of mDia1 (mammalian diaphanous-related formin 1) and polymerized actin in the phagocytic cup (101). Also, mDia1 binds directly to the microtubule-associated protein CLIP-170 at the phagocytic cup (102) and provides a link to the microtubule cytoskeleton required for CR-mediated phagocytosis (96, 97) (Figure 5).

## Phagosome Formation

Phagocytosis initiates when phagocytic receptors engage ligands on the particle to be ingested. Then, receptors activate signaling pathways that change the membrane composition and control the actin cytoskeleton, resulting in the formation of membrane protrusions for covering the particle. Finally, these membrane protrusions fuse at the distal creating a new vesicle that pinches out from the plasma membrane. This new vesicle containing the ingested particle is the phagosome.

During phagosome formation the membrane changes its lipid composition. These changes have been revealed by elegant fluorescence imaging techniques (3, 103), and involve the formation and degradation of different lipid molecules on the phagosome membrane in an orderly fashion. During Fcγ receptor-mediated phagocytosis, phosphatidylinositol-4,5-bisphosphate [PI(4,5)P_2_] initially accumulates at the phagocytic cup but then it declines rapidly (91). The decline in PI(4,5)P_2_ is important for particle internalization, probably by facilitating actin disassembly (104). The decline in PI(4,5)P_2_ is caused by the action of PI 3-K, which phosphorylates it to produce PI(3,4,5)P_3_ at the phagocytic cup (105). Reduction of PI(4,5)P_2_ in the membrane is also mediated by the action of PLCγ, which produces diacylglycerol (DAG) (91). DAG in turn, induces activation of PKCε for enhanced phagocytosis (92).

Together with the changes in lipid composition, the plasma membrane also changes by remodeling the actin cytoskeleton in order to generate the membrane protrusions that will cover the target particle. Important steps for pseudopodia formation are recognized. First, the cortical cytoskeleton gets disrupted. Second, pseudopodia are formed by F-actin polymerization. Third, at the base of the phagocytic cup, actin gets depolymerized while the membrane phagosome is sealed at the distal end to form the phagosome (15). When phagocytosis is initiated, the membrane-associated cortical cytoskeleton is altered by the action of coronins (F-actin debranching proteins) (106), and cofilin (107) and gelsolin (108) (F-actin-severing proteins). Coronin 1 concentrates at the nascent phagosome and debranches F-actin leaving linear fibers that can be severed by cofilin and gelsolin. The activity of these enzymes is controlled by their binding to phosphoinositides, such as PI(4,5)P_2_, resulting in their association with or separation from actin filaments (108, 109). Next, nucleation of new actin filaments, mediated by the actin-nucleating activity of the Arp2/3 protein complex, leads to pseudopodia formation. During FcγR-mediated phagocytosis, the GTPase Cdc42 and the lipid PI(4,5)P_2_ activate the proteins WASP (Wiskott-Aldrich syndrome protein) and N-WASP (110), which induce activation of Arp2/3 complex at the nascent phagocytic cup (111, 112). Different from this, during CR-mediated phagocytosis, actin polymerization is regulated by the GTPase Rho (113). Rho leads to activation of the Arp2/3 complex, via Rho kinase and myosin II (100). The Arp2/3 complex then produces branched actin-filament assembly at the phagocytic cup (100, 114). Rho also promotes accumulation of mDia1, which produces long straight actin filaments at the phagocytic cup (101, 114) (Figure 5). Together, these changes help extend membrane protrusions that completely cover the target particle.

The final step for phagosome formation involves fusion of the membrane protrusions at the distal end to close the phagosome. Just before the phagosome is completed, F-actin disappears from the phagocytic cup. It is thought that removal of actin filaments from the phagocytic cup may facilitate curving of the membrane around the particle (115). The mechanism for removing F-actin involves termination of actin polymerization and depolymerization of existing filaments. Both steps seem to be controlled by PI 3-K. Inhibition of this enzyme blocks actin depolymerization at the phagocytic cup and stops pseudopod extension (90). Activation of GTPases is necessary for stimulating the Arp2/3 complex during phagocytosis for actin polymerization (116). But, PI(3,4,5)P_3_, the product of PI 3-K can stimulate Rho-family GAPs (GTPase activating proteins), which cause deactivation of GTPases and in consequence prevents actin polymerization. In support of this model, it was found that inhibition of PI 3-K led to an increase of activated GTPases at the phagocytic cup (94, 116). In addition, the activity of PI-3K decreases the levels of PI(4,5)P_2_. This phospholipid activates the Arp2/3 complex, via WASP and N-WASP (110). Thus, its disappearance at the phagocytic cup (111, 112) promotes pseudopod extension (90).

It seems that myosins, actin-binding proteins (117, 118) use their contractile activity to facilitate phagosome formation. In macrophages, it was shown that class II, and IXb myosins were concentrated at the base of phagocytic cups, while myosin Ic increased at the site of phagocytic cup closure, and myosin V appeared after phagosome closure (119). During pseudopod extension, a tight ring of actin filaments moves from the bottom toward the top of the phagocytic cup squeezing the particle to be ingested (120). This contractile activity is dependent of myosin light-chain kinase (MLCK). Thus, myosin II activated by MLCK is required for the contractile activity of phagocytic cups (121). It seems that the squeezing action of the phagocytic cups pushes extra-particle fluid out of the phagosomes. Myosin X is also recruited to phagocytic cups in a PI 3-K-dependent manner, and seems to be important for pseudopod spreading during phagocytosis (122). At the same time, myosin Ic, a subclass of myosin I, concentrates at the tip of the phagocytic cup, implicating it in generating the contraction force that closes the opening of phagocytic cups in a purse-string-like manner (123). Myosin IX also appears in phagocytic cups similarly to myosin II (119, 123). Thus, it is believed that myosin IX is involved in the contractile activity of phagocytic cups. However, it is also possible that myosin IX functions as a signaling molecule for the reorganization of the actin cytoskeleton. This idea is based on the fact that class IX myosins contain a GTPase-activation-protein (GAP) domain that activates the GTPase Rho (124) involved in actin remodeling. Finally, myosin V appears on fully internalized phagosomes. Because class V myosins are involved in vesicular transport in other cell types (125), it is possible that myosin V is responsible for phagosome movement rather than formation of phagosomes (120). Video microscopy experiments have shown that newly formed phagosomes remain within the periphery of the cells for a while, hence it is likely that myosin V mediates the short-range slow movement of newly formed phagosomes (126). Consequently, the described roles of myosins during phagosome formation are: myosin II is involved in phagocytic cup squeezing, myosin X and myosin Ic are responsible for pseudopod extension and phagocytic-cup closing, respectively, myosin IX may activate Rho to direct actin remodeling, and myosin V controls the short-range movement of new phagosomes.

## Phagosome Maturation

Once internalized the new phagosome transforms its membrane composition and its contents, to become a new vesicle, the phagolysosome, that can degrade the particle ingested. This transformation is known as phagosome maturation, and consists of successive fusion and fission interactions between the new phagosome and early endosomes, late endosomes, and finally lysosomes (4, 127).

### Early Phagosome

**Figure 6:**
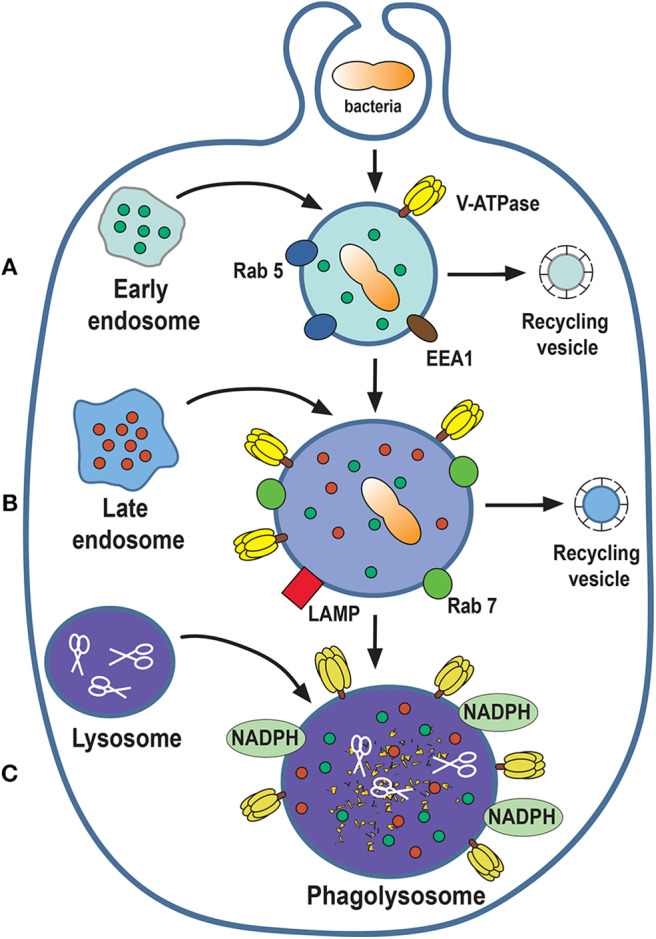
Phagosome maturation. The nascent phagosome gets transformed into a microbicidal vacuole, the phagolysosome, by sequential interactions with vesicles from the endocytic pathway. The process can be described in three stages of maturation: early **(A)**, late **(B)**, and phagolysosome **(C)**. In this process, composition of the membrane changes to include molecules that control membrane fusion, such as the GTPases Rab5 and Rab7. The phagolysosome becomes increasingly acidic by the action of a proton-pumping V-ATPase and acquires various degradative enzymes, such as cathepsins, proteases, lysozymes, and lipases (scissors). EEA1, early endosome antigen 1; LAMP, lysosomal-associated membrane protein; NADPH, nicotinamide adenine dinucleotide phosphate oxidase.

The new phagosome combines with early endosomes (3) in a process that involves membrane fusion events regulated by the small GTPase Rab5 (128, 129). Rab5 recruits the molecule EEA1 (early endosome antigen 1), promoting the fusion of the new phagosome with early endosomes (130). EEA1 functions as a bridge between early endosomes and endocytic vesicles (131), and promotes recruitment of other proteins, such as Rab7 (132, 133). Although, the new phagosome combines with several endosomes it does not increase in size because at the same time vesicles, named recycling endosomes, are removed from the phagosome (Figure 6).

### Late Phagosome

As phagosome maturation proceeds, Rab5 is lost, and Rab7 appears on the membrane (133). Then, Rab7 mediates the fusion of the phagosome with late endosomes (134). At the same time, there is an accumulation of V-ATPase molecules on the phagosome membrane. This V-ATPase is responsible for the acidification (pH 5.5–6.0) of the phagosome interior by translocating protons (H^+^) into the lumen of the phagosome (135, 136) (Figure 6). Also, lysosomal-associated membrane proteins (LAMPs) and luminal proteases (cathepsins and hydrolases) are incorporated from fusion with late endosomes (4, 127) (Figure 6).

### Phagolysosome

At the last stage of phagosome maturation, phagosomes fuse with lysosomes to become phagolysosomes (3). The phagolysosome is the fundamental microbicidal organelle, equipped with sophisticated mechanisms for degrading microorganisms. First, phagolysosomes are very acidic (pH as low as 4.5) due to the accumulation of many V-ATPase molecules on their membrane (136). The phagolysosome membrane also presents the NADPH oxidase complex, that is responsible for producing reactive oxygen species (ROS), such as superoxide (O2^−^) (137, 138). Superoxide dismutates to H_2_O_2_, which can in turn react with Cl^−^ ions to form hypochlorous acid, a very potent microbicidal substance. This last reaction is catalyzed by the enzyme myeloperoxidase (139). In addition, the phagolysosome contains several hydrolytic enzymes, such as cathepsins, proteases, lysozymes, and lipases, which contribute to degrade ingested microorganisms (135) (Figure 6).

## Phagocytosis-Associated Responses

Phagocytosis is not an isolated cell response. It usually occurs together with other cell responses, including formation of reactive oxygen species (ROS) (140, 141), secretion of pro-inflammatory mediators (142), degranulation of anti-microbial molecules (143, 144), and production of cytokines (142). Cell responses associated to phagocytosis can be controlled by parallel signaling pathways triggered by the same phagocytic receptors. For instance, antibody-dependent phagocytosis in monocytes is controlled by PKC, independently of PI 3-K and ERK (145). However, in the same monocytes, antibody stimulation induces cytokine production via PI 3-K and ERK (145). Phagocytosis and associated cell responses can also be controlled by partially overlapping signaling pathways. For instance, antibody-dependent phagocytosis, in macrophages involves the signaling molecules Syk, PI 3-K, PKC, and ERK, but it is independent of an increase in cytosolic calcium concentration (146, 147). In contrast, in neutrophils production of ROS also involves Syk, PI 3-K, PKC, and ERK, but it is dependent on cytosolic calcium (148). Also, in macrophages different PKC isoforms seem to be required either for phagocytosis, or for production of ROS. The isoforms PKCδ and PKCε are involved in regulation of phagocytosis, while PKCα is involved in regulation of ROS production (92). These observations suggest that particular Fcγ receptors can trigger diverse signaling pathways for specific cell responses (55). In support of this idea, in neutrophils in was found that FcγRIIa and FcγRIIIb signal differently for phagocytosis (149), and also for neutrophil extracellular trap (NET) formation (150).

## Phagocytosis Efficiency

Most phagocytes have relatively low levels of phagocytosis at resting conditions. However, during inflammation, phagocytes are exposed to a variety of activating stimuli, which increase phagocytosis efficiency. These stimuli include bacterial products, cytokines, and inflammatory mediators. The signaling induced by these stimuli leads to increased stimulation of molecules involved in phagocytosis. For example, leukotriene B4 increases Syk activation and in consequence antibody-dependent phagocytosis (151). Similarly, the activity of PI 3-K and/or ERK, which are essential enzymes for efficient phagocytosis (83), can be enhanced by the bacterial peptide fMLF (152), granulocyte colony-stimulating factor (153), leukotrienes (154), and cytokines such as interleukin 8 (IL-8) (155).

Phagocytosis efficiency can also be regulated by cell differentiation. For example, monocytes have a lower phagocytic capacity than neutrophils and macrophages, but can enhance their phagocytic capacity upon cell differentiation (1, 156). The capacity of monocytes to phagocytize diverse targets changes with their state of differentiation. IgG-opsonized particles are phagocytized better by mature macrophages than by undifferentiated monocytes (83). Similarly, the efficiency of complement-mediated phagocytosis depends on monocyte differentiation (157, 158). How the process of monocyte-to-macrophage differentiation enhances phagocytic capacity is still unknown. It is possible that during cell differentiation the molecular machinery for phagocytosis gets rearranged. In support of this idea, it was found that in monocytes phagocytosis signaling requires PKC, but it does not use PI 3-K and ERK (145). However, during monocyte-to-macrophage differentiation the enzymes PI 3-K and ERK are recruited in an orderly fashion for efficient phagocytosis (159). Similarly, PLA2 is also implicated in regulation of phagocytosis. During phagocytosis, various PLA2 isoforms participate in releasing arachidonic acid from membrane triglyceride lipids. In monocytes, a calcium-independent PLA2, under PKC control is involved in phagocytosis (160, 161), while in macrophages, a calcium-dependent PLA2, under ERK and p38MAPK control is involved (162). Thus, during monocyte-to-macrophage differentiation important signaling enzymes are reorganized in order to achieve enhanced phagocytosis.

## Conclusion

Phagocytosis is a fundamental process for the ingestion and elimination of microbial pathogens and apoptotic cells. All types of cells can perform phagocytosis, but specialized cells called professional phagocytes do it much more efficiently. Phagocytosis is vital, not only for eliminating microbial pathogens, but also for tissue homeostasis. Because there are different types of phagocytic cells and they can ingest a vast number of different targets, it is evident that phagocytosis involves diverse mechanisms. We have presented the main steps of phagocytosis as performed by professional phagocytes and in response mainly to Fcγ receptors. For other phagocytic receptors, we are just beginning to describe the signaling pathways they use to activate phagocytosis. Today, we have a better understanding on the process of phagosome maturation, but there are still many gaps in our knowledge of the signaling pathways regulating this process. Similarly, the resolution of the phagolysosome, after degradation of the ingested particle, is a topic that requires further research. Many important questions remain unsolved. For example, how different phagocytic receptors on the same cell work together? and what is the role different phagocytes in tissue homeostasis? An improved understanding of phagocytosis is essential for future therapeutics related to infections and inflammation.

## Author Contributions

EU-Q prepared the reference list, made the figures and reviewed the manuscript. CR conceived the issues which formed the content of the manuscript and wrote the manuscript.

## Conflict of Interest

The authors declare that the research was conducted in the absence of any commercial or financial relationships that could be construed as a potential conflict of interest.
